# One Stone, Three Birds: Multifunctional Nanodots as “Pilot Light” for Guiding Surgery, Enhanced Radiotherapy, and Brachytherapy of Tumors

**DOI:** 10.1021/acscentsci.3c00994

**Published:** 2023-10-17

**Authors:** Ze Wang, Dongzhou Wang, Xiaojun Ren, Zhongshan Liu, Annan Liu, Xingchen Li, Lin Guan, Yannan Shen, Shunzi Jin, Andrei V. Zvyagin, Bai Yang, Tiejun Wang, Quan Lin

**Affiliations:** † State Key Laboratory of Supramolecular Structure and Materials, College of Chemistry, Jilin University, Changchun 130012, P. R. China; ‡ Department of Radiation Oncology, The Second Affiliated Hospital of Jilin University, Changchun 130041, P. R. China; § NHC Key Laboratory of Radiobiology, School of Public Health, Jilin University, Changchun 130021, P. R. China; ∥ Australian Research Council Centre of Excellence for Nanoscale Biophotonics, Macquarie University, Sydney, NSW 2109, Australia; ⊥ Institute of Biology and Biomedicine, Lobachevsky Nizhny Novgorod State University, 603105 Nizhny Novgorod, Russia

## Abstract

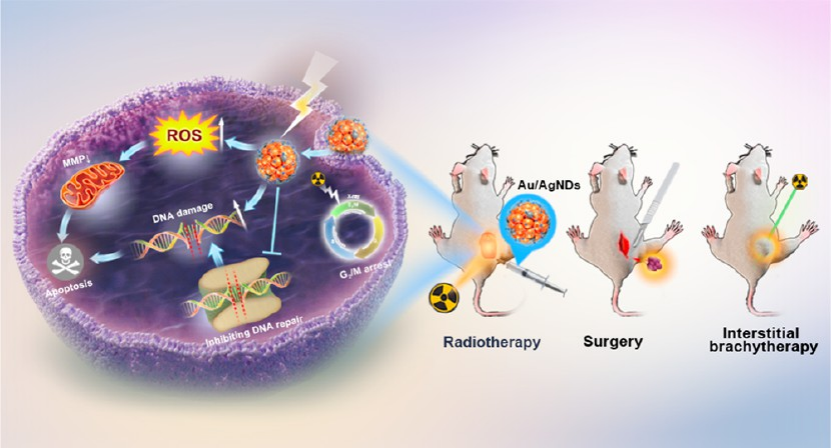

Surgery, radiotherapy (RT), and brachytherapy are crucial treatments for localized deep tumors. However, imprecise tumor location often leads to issues such as positive surgical margins, extended radiotherapy target volumes, and radiation damage to healthy tissues. Reducing side effects in healthy tissue and enhancing RT efficacy are critical challenges. To address these issues, we developed a multifunctional theranostic platform using Au/Ag nanodots (Au/AgNDs) that act as a “pilot light” for real-time guided surgery, high-efficiency RT, and brachytherapy, achieving a strategy of killing three birds with one stone. First, dual-mode imaging of Au/AgNDs enabled precision RT, minimizing damage to adjacent normal tissue during X-ray irradiation. Au/AgNDs enhanced ionizing radiation energy deposition, increased intracellular reactive oxygen species (ROS) generation, regulated the cell cycle, promoted DNA damage formation, and inhibited DNA repair in tumor cells, significantly improving RT efficacy. Second, in brachytherapy, precise guidance provided by dual-mode imaging addressed challenges related to non-visualization of existing interstitial brachytherapy and multiple adjustments of insertion needle positions. Meanwhile, the effect of brachytherapy was improved. Third, the excellent fluorescence imaging of Au/AgNDs accurately distinguished tumors from normal tissue, facilitating their use as a powerful tool for assisting surgeons during tumor resection. Taken together, our multifunctional theranostic platform offers real-time guidance for surgery and high-efficiency RT, and improves brachytherapy precision, providing a novel strategy and vision for the clinical diagnosis and treatment of cancer.

## Abstract 2

We developed a multifunctional theranostic platform that acts as a “pilot light” for guiding surgery, high-efficiency RT, and brachytherapy, achieving a strategy of killing three birds with one stone.

## 1 Introduction

Malignant tumors continue to be a leading cause of death worldwide. In clinical practice, surgical excision and radiotherapy (RT) are effective local treatments for tumors. However, both modalities have their limitations. Surgical excision often involves sacrificing adjacent normal tissue to ensure a safe boundary and achieve a negative margin.^1^ RT, an effective treatment for tumors, relies on high-energy ionizing radiation and reactive oxygen species (ROS) to damage intracellular DNA double strands and effectively treat tumors.^2,3^ Nonetheless, RT faces challenges, such as low energy absorption in tumor tissue and difficulties in killing radioresistant tumor cells.^4^ Moreover, existing radiosensitizers, like conventional chemotherapy molecules or complexes, often come with significant side effects and limited efficacy, hindering their widespread use.^5−8^ The development of high-efficiency and low-toxicity radiosensitizers is the focus of the current research. In addition, due to the difficulty in accurately locating the tumor, ionizing radiation used to kill tumors may damage adjacent normal tissue, resulting in serious toxicities of the organism.^9^ Brachytherapy, a method involving the placement of a radiation source within the tumor area, complements RT and enhances precision.^10^ However, interstitial brachytherapy is an invasive procedure with high technical complexity,^11^ making accurate needle placement challenging without real-time imaging guidance. Given these challenges, an “all-in-one” material that integrates real-time imaging-guided treatment process (such as surgery, RT and brachytherapy) and improving the efficiency of RT is urgently needed for precise diagnosis and treatment of tumors.

In recent years, nanomaterials have shown great promise in the biomedical field, particularly in cancer diagnosis and enhanced RT.^12−14^ Among them, gold nanoparticles (AuNPs) have garnered significant attention from researchers due to their ultrasmall size, easy surface functionalization, and excellent biocompatibility.^15,16^ On the one hand, AuNPs, high Z materials, have strong X-ray or γ-ray attenuation capability, making them ideal radiosensitizers to concentrate radiation energy within tumors and improve radiotherapeutic efficiency.^17−19^ On the other hand, the unique optical properties and good X-ray attenuation ability of AuNPs make them applicable to accurate tumor imaging, such as fluorescence (FL) imaging and computed tomography (CT).^20,21^ Furthermore, researchers have found that silver nanoparticles (AgNPs) have also demonstrated the ability to enhance RT sensitivity.^22^ AgNPs can increase intracellular ROS, activate oxidative stress, regulate the cell cycle, induce cell apoptosis, and exhibit excellent antitumor ability, making them increasingly utilized in the biomedical field.^23−27^ Therefore, the development of an effective and simple method to assemble AuNPs and AgNPs into a single multifunctional spherical nanoparticle holds significant potential for real-time imaging-guided treatment and enhanced RT efficacy.

**Scheme 1:**
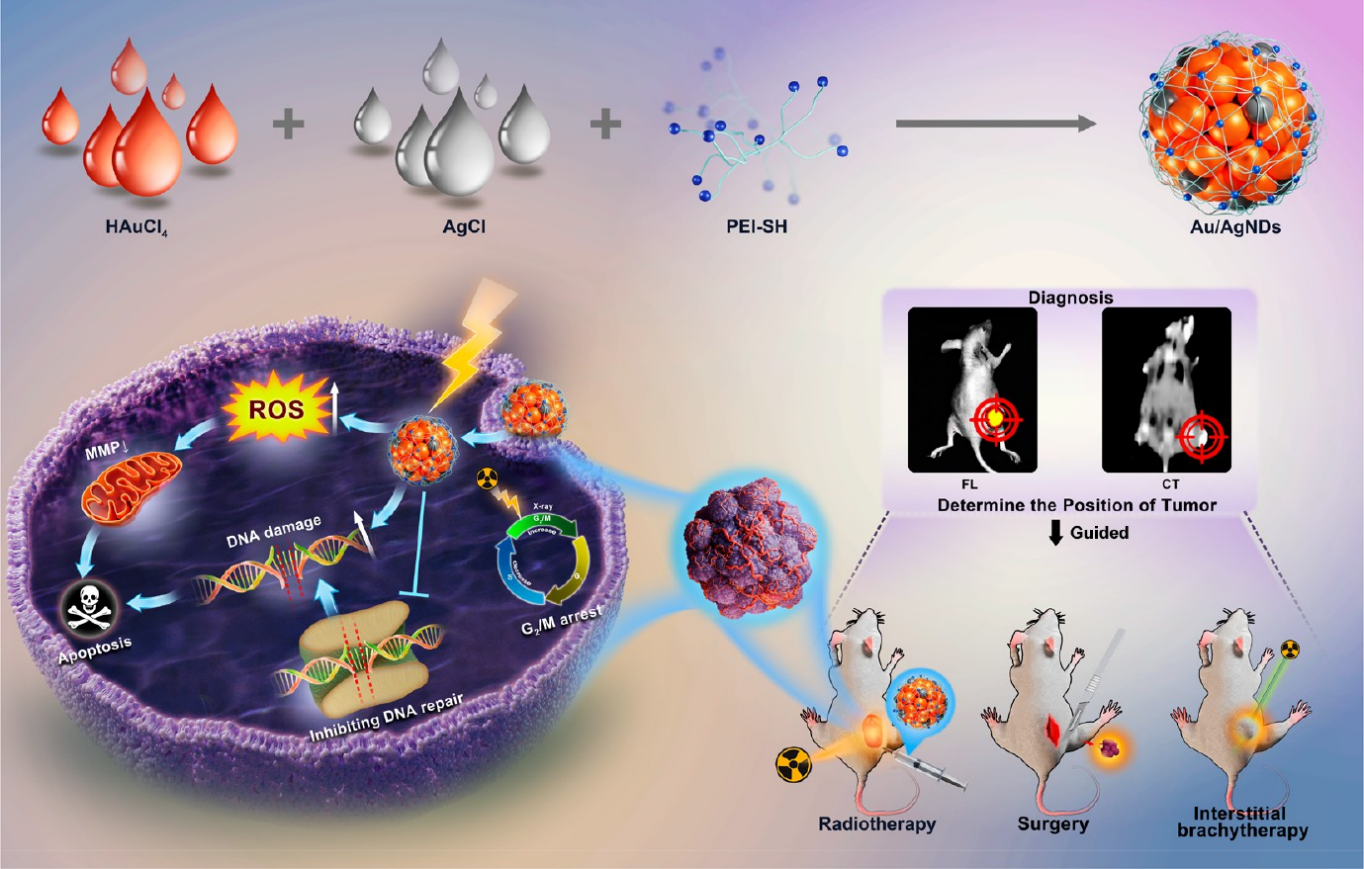
Schematic Illustration of Synthesis of Au/AgNDs Used for Dual-Mode Imaging-Guided Surgery, Enhanced Radiotherapy, and Brachytherapy of Tumors

To solve the aforementioned challenges, we designed excellent nanoprobe and radiosensitizer Au/Ag nanodots (Au/AgNDs) (Scheme 1). Compared with AuNDs, Au/AgNDs not only enhance the effect of FL imaging but also improve the effectiveness of RT. The ability and mechanism of Au/AgNDs for sensitized RT of tumor cells were then tested through investigation of cell viability, colony formation, the cell apoptosis effect, ROS generation, cell cycle progression, DNA damage, and DNA repair. Moreover, FL/CT dual-mode imaging of Au/AgNDs can serve as a “pilot light” to enhance the accuracy of tumor diagnosis. By utilizing both imaging modes in conjunction, a more precise determination of the location of the tumor is achieved. The excellent dual-mode imaging capability allows for accurate identification of the tumor location and real-time imaging guidance during surgery, RT, and brachytherapy.

## 2 Results and Discussion

### 2.1 Synthesis Strategy and Characterization of Au/AgNDs

In this work, multifunctional Au/AgNDs were synthesized and used for dual-mode imaging-guided surgery, enhanced RT, and brachytherapy of tumors. Briefly, the ligand mercaptosylated polyethylenimine (SH-PEI) was synthesized by amidation reaction using 3-mercaptopropionic acid (MPA) and PEI. Then, using the prepared SH-PEI as the ligand, HAuCl_4_ and AgNO_3_ were introduced into the system to synthesize Au/AgNDs. To confirm the successful synthesis and assess the structure of the Au/AgNDs, we analyzed their infrared spectra. The infrared spectra of PEI, SH-PEI, and Au/AgNDs are shown in Figure S1. In the infrared spectrum of SH-PEI, it can be seen that characteristic peaks at 2488 cm^–1^ (attributed to −SH) and 1394 cm^–1^ (corresponding to amide bonds) can be seen, confirming the successful connection between MPA and PEI. The ligand SH-PEI can form the Au–S bond with Au. In the infrared spectrogram of Au/AgNDs, the characteristic peak of sulfhydryl at 2488 cm^–1^ disappeared, indicating that Au/AgNDs with SH-PEI as the ligand have been successfully synthesized.

**Figure 1:**
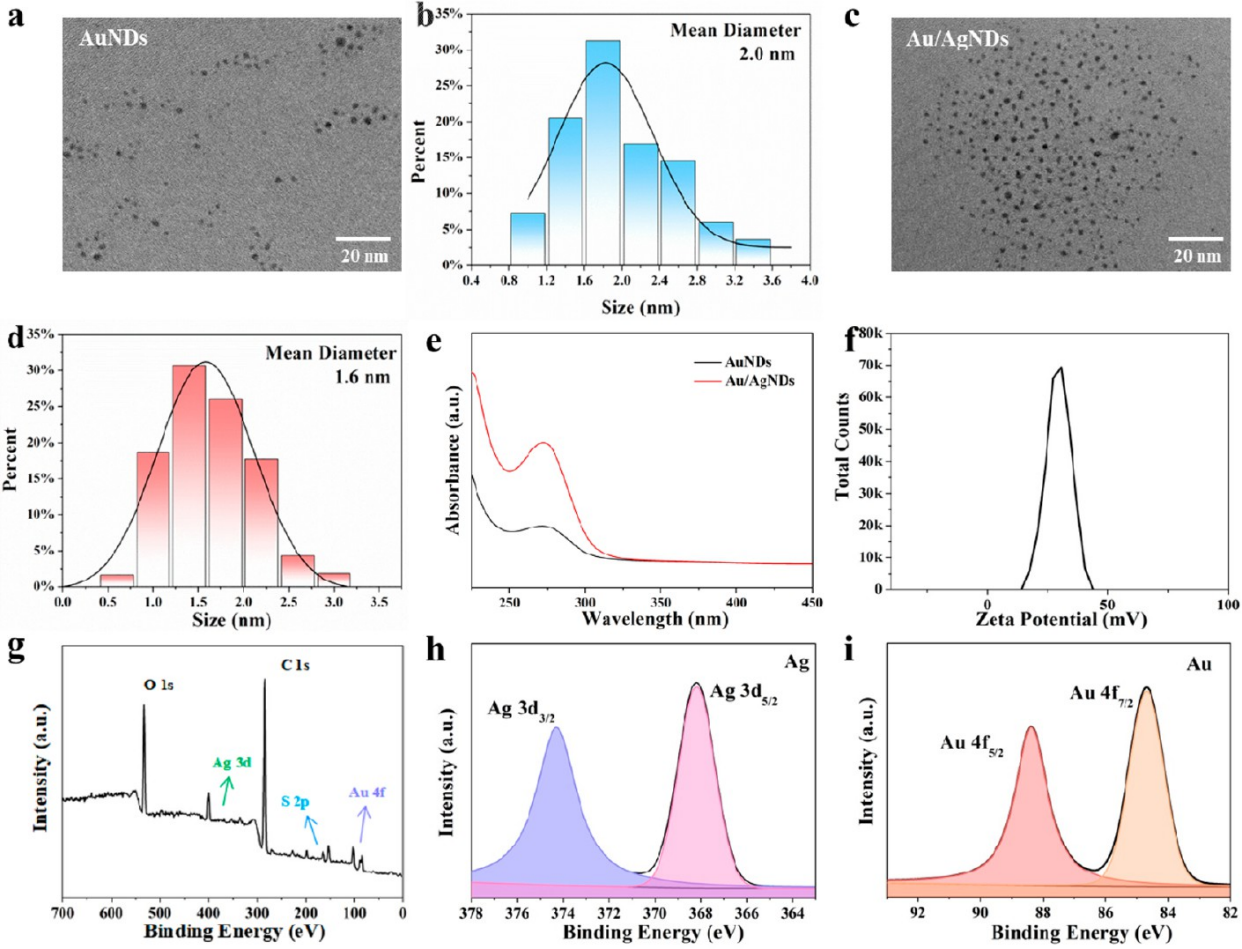
Characterization of the structure and morphology of Au/Ag nanodots: (a) TEM image and (b) size distribution histogram of AuNDs. (c) TEM image and (d) size distribution histogram of Au/AgNDs. (e) Ultraviolet–visible spectra of AuNDs and Au/AgNDs. (f) Zeta potential of Au/AgNDs. (g) XPS survey spectrum of Au/AgNDs. High-resolution XPS spectra of Ag 3d (h) and Au 4f (i) of Au/AgNDs.

Doping precious metals has been reported as an effective method to control material structure and enhance physicochemical properties, such as fluorescence intensity and stability. To optimize the structure and performance of AuNDs, Au/AgNDs were synthesized. Initially, the morphology of the AuNDs and Au/AgNDs was investigated by transmission electron microscope (TEM). As can be seen from Figure 1a and b, the AuNDs show a spherical shape with an average size of 2.0 nm. The Au/AgNDs still maintain good spherical morphology, and the average size (1.6 nm) is slightly smaller than that of the AuNDs (Figure 1c and d). In the UV absorption spectrum, the intensity of the characteristic peak representing metal–sulfhydryl binding at about 273 nm increased (Figure 1e). This indicates a stronger binding between the metal inside the nanodots and the external ligand, resulting in enhanced overall stability of the Au/AgNDs. The surface charge of nanomaterials plays a crucial role in the cellular uptake, especially in the case of cancer cells. Therefore, to facilitate efficient internalization by cancer cells, nanomaterials are often designed with a positive charge.^28^ The zeta potentials of Au/AgNDs were determined by dynamic light scattering (DLS) (Figure 1f). Au/AgNDs synthesized by using SH-PEI as the ligand have a significant positive charge on the surface. The high positive charge and small size of Au/AgNDs allow them to readily interact with the negatively charged phospholipid bilayer on the cell membrane, facilitating cell endocytosis.

The elemental composition and valence states of Au/AgNDs were characterized by X-ray photoelectron spectroscopy (XPS). As shown in the XPS survey spectrum, Au/AgNDs contain characteristic peaks of the O 1s, C 1s, Ag 3d, S 2p, and Au 4f (Figure 1g). The characteristic peaks at 374.3 and 368.2 eV were assigned to Ag 3d_3/2_ and Ag 3d_5/2_ (Figure 1h), respectively, indicating the coexistence of Ag (I) and Ag (0) in the Au/AgNDs.^29,30^ The characteristic peaks appearing at 88.4 and 84.7 eV were assigned to Au 4f_5/2_ and Au 4f_7/2_, indicating that Au (0) and Au (I) were both contained in the Au/AgNDs^31^ (Figure 1i). The presence of Au (0) promotes the nucleation of nanodots, while Au (I) is conducive to the formation of stable metal–sulfhydryl (Au–S) covalent bonds with the ligand. The stable binding of Au–S bonds enables continuous energy transfer between the ligand and metal, leading to stable and bright fluorescence properties of Au/AgNDs. The characteristic peaks of S 2p appear at 164.6 and 163.5 eV (Figure S2). In addition, energy-dispersive spectroscopy (EDS) can further indicate that the material contains Au, Ag, and S elements (Figure S3). All above results supported the formation of the stable structure of Au/AgNDs.

### 2.2 Dual-Mode Imaging of Au/AgNDs in Vitro

Contrast agents play a crucial role in distinguishing lesion sites and providing accurate information. However, the commonly used CT contrast agents in clinical settings are small-molecule drugs with inherent limitations. For instance, iodine small-molecular agents, frequently employed in clinical practice, suffer from issues like short blood circulation time, limited modification possibilities, and potential toxic and side effects.^32,33^ Furthermore, various fluorescent probes, such as carbon dots and down-conversion nanoparticles, have been employed for FL imaging. Nevertheless, these fluorescent probes face challenges such as fluorescent bleaching, high toxicity, and hydrophobicity.^34^ Additionally, most clinical contrast agents are only suitable for single-mode imaging, while developing contrast agents with multiple imaging functions for cancer diagnosis often involves complex structures and synthetic processes.^35,36^ Based on the above challenges, Au/AgNDs with an excellent FL/CT dual-mode imaging effect were designed.

**Figure 2:**
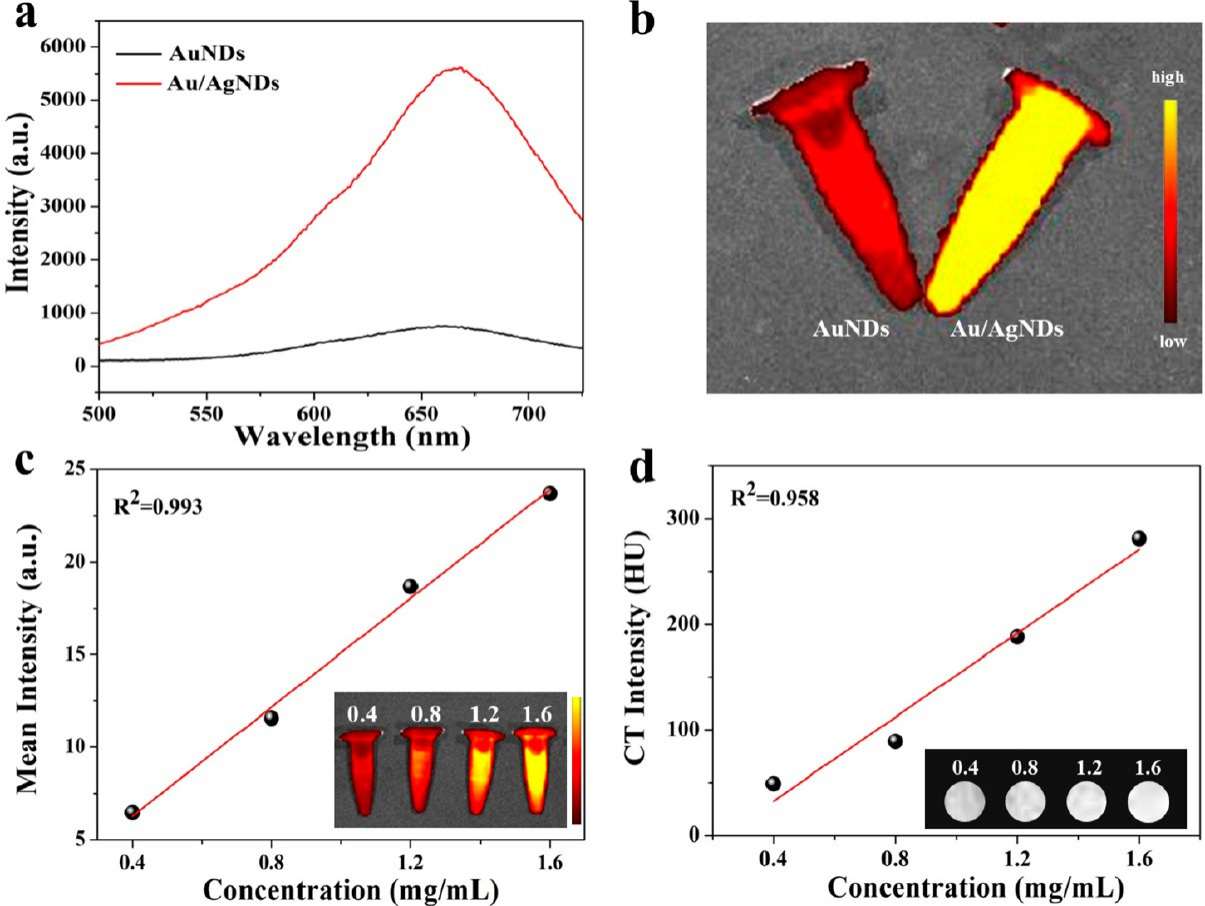
Enhanced FL imaging and CT imaging of Au/AgNDs. (a) Fluorescence spectra of AuNDs and Au/AgNDs. (b) FL imaging photographs of AuNDs and Au/AgNDs. (c) FL and (d) CT intensity trend of Au/AgNDs at different concentrations. Inset: FL and CT images at different concentrations of Au/AgNDs.

AgNPs have been shown to reduce the fluorescence lifetime while enhancing the fluorescence intensity of fluorescent molecules by increasing the radiation decay rate.^37−40^ The in vitro dual-mode imaging ability of Au/AgNDs was examined. As shown in Figure 2a, the fluorescence intensity of Au/AgNDs was significantly improved, which was 6 times that of AuNDs. The difference in fluorescence intensity between AuNDs and Au/AgNDs can be visually seen in Figure 2b. The existence of AgNPs reduces the fluorescence lifetime of the nanodots from 11.51 to 9.073 μs (Figure S4), which is due to an increase in the radiation decay rate of the nanodots. This indicates that the excited state time of nanodots was shortened, leading to enhanced fluorescence intensity. Additionally, we observed a gradual shift in the wavelength of Au/AgNDs toward the near-infrared region. Moreover, it was found that the FL (Figure 2c) and CT (Figure 2d) signals gradually enhanced with an increase in concentration, indicating their good FL/CT imaging ability. The degree of Au/AgNDs enrichment can also be quantified based on the imaging effect. Overall, the synthesized Au/AgNDs nanosystem has the potential for in vivo FL/CT dual-mode imaging and can be used for accurate imaging of diseases.

The fluorescence stability of biomaterials is crucial for in vivo FL imaging. First, the fluorescence stability of Au/AgNDs was investigated in the physiologically relevant pH range (4–10).^41^ The results demonstrated that the fluorescence intensity of Au/AgNDs remained relatively stable within this pH range, indicating excellent pH stability (Figure S5). The ionic stability of Au/AgNDs was assessed in the presence of common cations (K^+^, Na^+^, NH_4_^+^, Ca^2+^). As shown in Figure S6, even with high concentrations of interfering ions, the fluorescence intensity of Au/AgNDs remained stable, demonstrating great ionic stability. Additionally, we investigated the light stability of the Au/AgNDs solution by exposing it to ultraviolet light for 3 h. As shown in Figure S7, the fluorescence intensity of Au/AgNDs hardly changes, and no fluorescence bleaching occurs, indicating that Au/AgNDs have good light stability. The fluorescence intensity of Au/AgNDs remained well-maintained, even after 21 days (Figure S8). The fluorescence stability of Au/AgNDs is attributed to the Au–S bond, which firmly binds the ligand to the nanodots and protects the metal core of the nanodots. In conclusion, the prepared Au/AgNDs serve as an excellent and long-lasting fluorescent probe for in vivo applications. The formation of Au/AgNDs by doping precious metals allows us to effectively control the properties of the material, resulting in improved fluorescence intensity and stability for potential applications in various fields.

### 2.3 Biocompatibility and Cellular Uptake in Vitro

**Figure 3:**
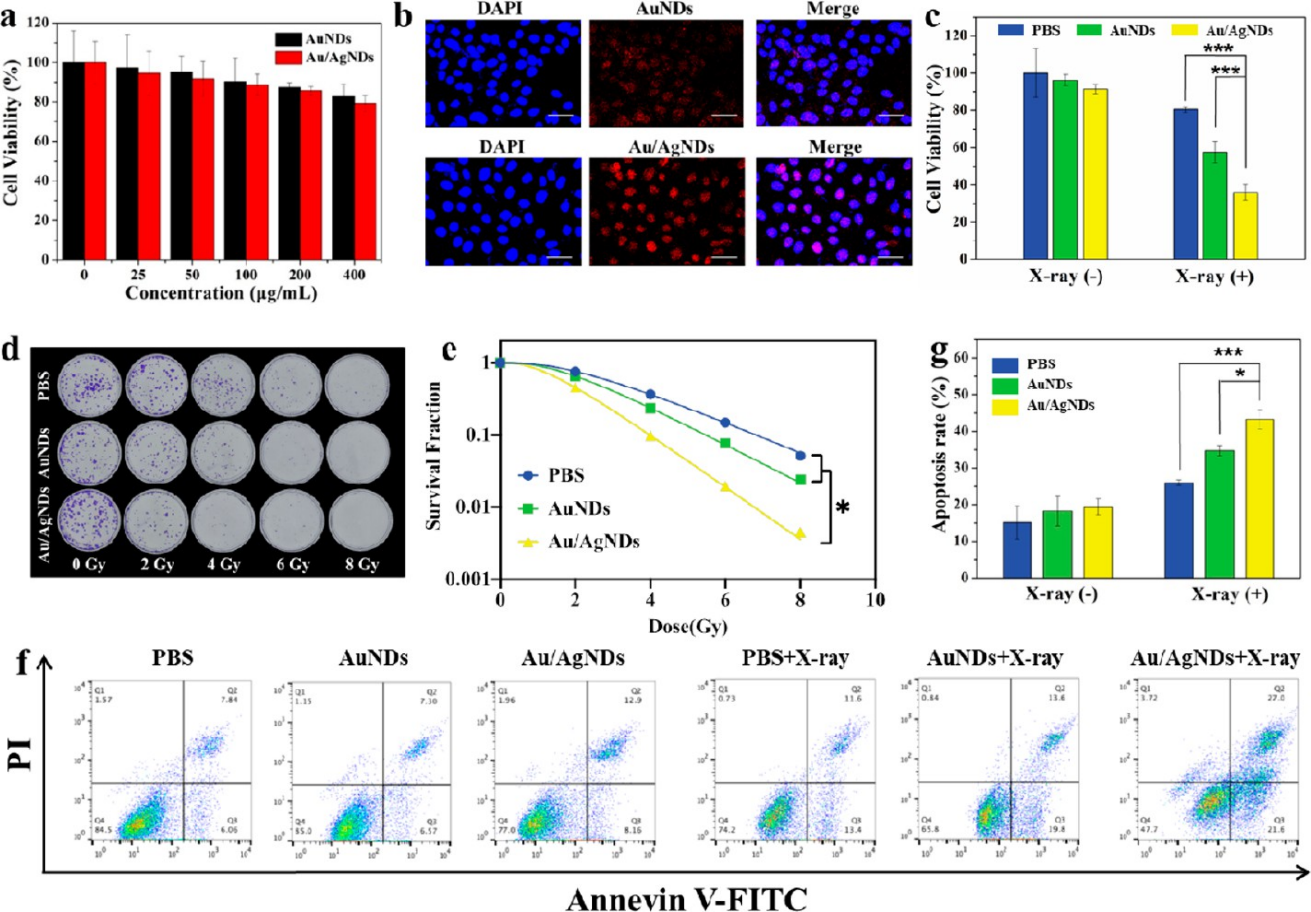
Biocompatibility, cellular uptake, and radiotherapy effect in vitro. (a) Effect of AuNDs and Au/AgNDs on the viability of HeLa cells at different concentrations for 24 h. (b) In vitro cell uptake by HeLa cells cultured with AuNDs or Au/AgNDs, as detected by CLSM. Scale bar: 40 μm. (c) Cell viability of HeLa cells incubated with PBS, AuNDs, or Au/AgNDs with/without X-ray (6 Gy). (d) Representative images of colony formation of HeLa cells after different doses of irradiation exposure. (e) Colony formation curves of HeLa cells after different treatments. (f) Percentage of apoptotic cells after incubation with PBS, AuNDs, or Au/AgNDs with/without X-ray (6 Gy). (g) Flow cytometric analysis of HeLa cells after incubation with PBS, AuNDs, or Au/AgNDs with/without X-ray (6 Gy).

Excellent biocompatibility is a prerequisite for the application of biomaterials in vivo. The cell biocompatibility of the AuNDs and Au/AgNDs was evaluated by a cell counting kit 8 (CCK8) assay. First, L929 cells were co-cultured with AuNDs or Au/AgNDs in gradient concentration (0, 100, 200, 300, and 400 μg/mL) for 24 h; the survival rate remained above 90%, verifying the biocompatibility of the materials (Figure S9). In addition, AuNDs or Au/AgNDs with different mass concentrations (0, 25, 50, 100, 200, and 400 μg/mL) were co-cultured with HeLa cells for 12 h (Figure S10) and 24 h (Figure 3a). Even after a long time of co-culture with 200 μg/mL AuNDs or Au/AgNDs, the HeLa cell survival rate remained above 80%, indicating that the nanodots had good biocompatibility, so the concentration of the nanodots in the subsequent cell experiments was 200 μg/mL.

The nanodots exhibited excellent fluorescence properties, allowing us to visually verify their cellular uptake by using confocal fluorescence microscopy. As seen from Figure 3b, AuNDs and Au/AgNDs showed good cellular uptake. Obviously, Au/AgNDs exhibited brighter red fluorescence, and the better FL imaging effect makes them more suitable for in vivo application as fluorescent probes.

### 2.4 Radiotherapy Effect in Vitro

Due to the poor efficacy and side effects of radiosensitizers used in clinical practice, Au/AgNDs with high efficiency of RT and low toxicity were designed. To verify the radiosensitization of the Au/AgNDs in vitro, the CCK8 experiment was performed. Cells were co-cultured with phosphate buffered saline (PBS), AuNDs, or Au/AgNDs for 12 h and then exposed to X-ray radiation (6 Gy) to investigate the cell survival rates for each group. As seen from Figure 3c, the survival rate of cells in the PBS+X-ray group was 80.42% ± 1.72%. Upon treatment with AuNDs+X-ray, the cell survival rate decreased to 57.55% ± 5.84%. Ulteriorly, the survival rate of the cells treated with Au/AgNDs+X-ray was significantly reduced to 35.95% ± 4.24%. To quantify the radiosensitization effect of Au/AgNDs, a cloning formation assay was performed. As irradiation doses increase, AuNDs and Au/AgNDs reduce the survival fraction of cells to a large extent (Figure 3d). Obviously, the inhibition ability of the Au/AgNDs group on colony formation was better than the AuNDs group. By fitting the cell survival curves, the sensitization enhancement ratio (SER) of AuNDs and Au/AgNDs was calculated to be 1.16 and 1.67, respectively, surpassing those of most metal-based radiosensitizers (Figure 3e, Table S1).

Flow cytometry was processed to detect whether Au/AgNDs can promote apoptosis and further provide evidence for outstanding anticancer ability. After X-ray irradiation, the apoptosis induced by Au/AgNDs was significantly higher than that induced by PBS and AuNDs (Figure 3f and g). This observation indicates that Au/AgNDs combined with X-ray irradiation effectively enhance RT sensitivity by promoting cell apoptosis, demonstrating an excellent RT effect.

### 2.5 Radiotherapy Enhancement Mechanism

**Figure 4:**
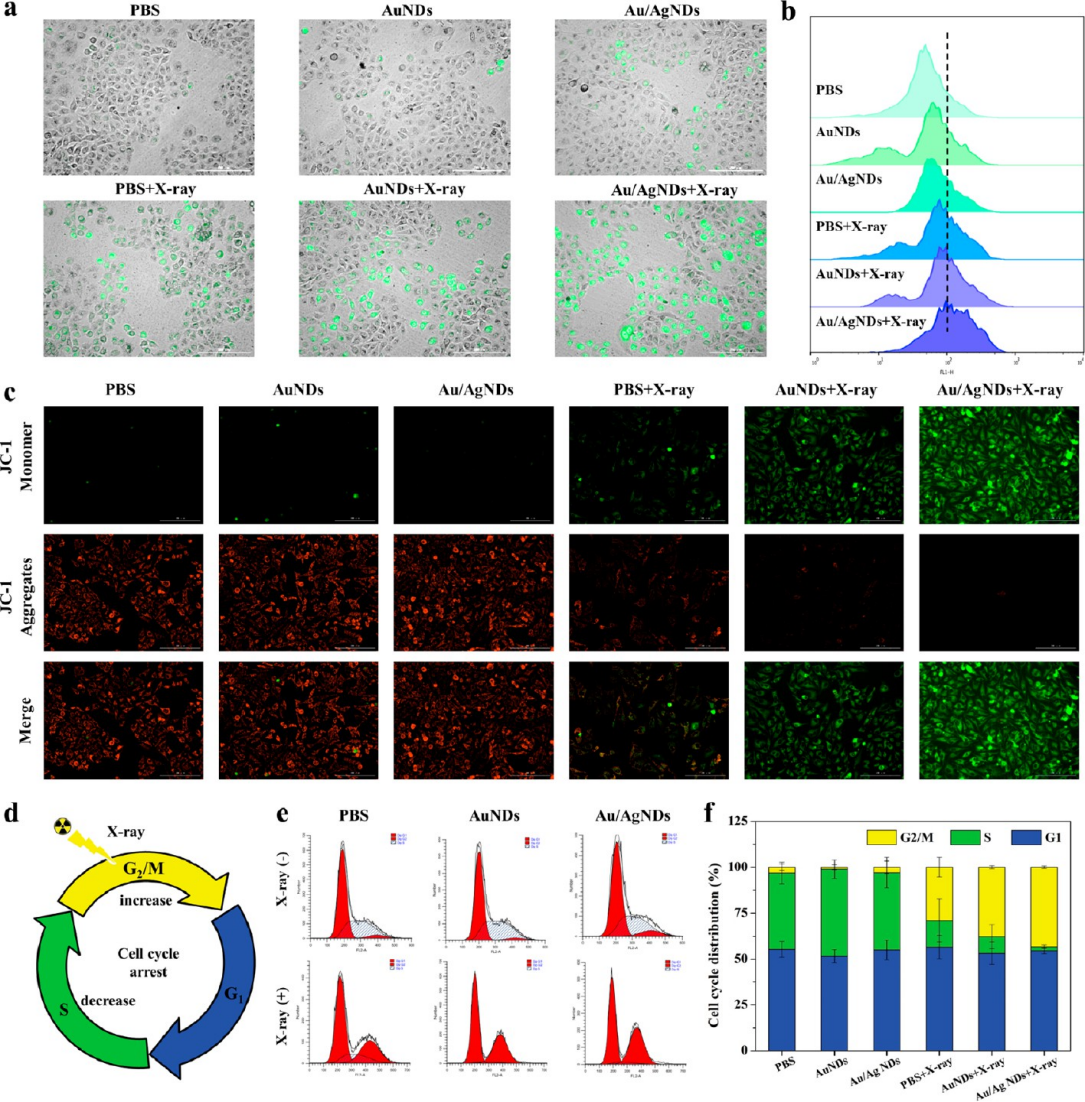
Mechanistic investigation of Au/AgNDs-induced radiosensitization. (a) Fluorescence microscope images of intracellular ROS detection in HeLa cells treated with PBS, AuNDs, or Au/AgNDs with/without X-ray (6 Gy), followed by staining with DCFH-DA (scale bar: 200 μm). (b) Flow cytometric assay in HeLa cells after incubation with PBS, AuNDs, or Au/AgNDs with/without X-ray (6 Gy). (c) Fluorescence microscope images of the mitochondrial membrane potential stained with JC-1 (scale bar: 200 μm). (d) Schematic diagram of nanosystem and X-ray irradiation enhanced G2/M phase arrest. (e, f) Cell cycle analysis and percentage (at different stages) of PBS, AuNDs, or Au/AgNDs with/without X-ray (6 Gy).

AuNPs, high Z materials, have strong X-ray or γ-ray attenuation ability and can be used as radiosensitizers to precipitate radiation energy in tumors and improve RT efficacy. When AuNPs are exposed to ionizing radiation, they efficiently absorb photons, leading to the emission of various electrons. These electrons can directly damage DNA or indirectly interact with H_2_O, generating ROS that cause DNA damage and induce cell apoptosis.^42^ Our research has confirmed that Au/AgNDs exhibit a superior RT effect compared to AuNDs. In the following discussion, we explore the mechanisms responsible for this enhancement:

Ionizing radiation interacts with intracellular H_2_O to generate ROS that causes damage to the organism. The accumulation of ROS can induce oxidative damage to biomacromolecules and mitochondria, thus inducing programmed cell death.^43,44^ Radiosensitizers can increase ROS production under X-ray irradiation, and previous studies have demonstrated that AgNPs induce an increase in intracellular ROS in various cell types.^23,27,45^ We used 2,7-dichlorodihydrofluorescein diacetate (DCFH-DA) as a fluorescent probe to quantify the ROS level in the PBS, AuNDs, and Au/AgNDs groups after X-ray irradiation. It can be seen more intuitively from Figure 4a that the ROS level in all groups was increased after X-ray irradiation, and more ROS can be generated after Au/AgNDs+X-ray treatment. Specifically, we used flow cytometry to quantitatively analyze the amount of ROS in each group; the ROS level in the Au/AgNDs+X-ray group was 1.20 times higher than that in the AuNDs+X-ray group and 1.50 times higher than that in the PBS+X-ray group (Figure 4b). In conclusion, the massive generation of ROS is the main reason for the enhanced RT effect of Au/AgNDs combined with X-ray.

When mitochondria are exposed to ionizing radiation and ROS, their membrane structure and permeability are affected, leading to a reduction in the potential difference between the inner and outer sides of the mitochondrial membrane and initiating programmed cell death.^46,47^ JC-1 is a fluorescent probe for detecting mitochondrial membrane potential. High mitochondrial membrane potential causes JC-1 to form red fluorescent polymers (*J*-aggregates), while a decrease in potential leads to the disaggregation of JC-1 into green fluorescent monomers. JC-1 was used to quantify mitochondrial membrane potential in PBS, AuNDs, and Au/AgNDs after X-ray irradiation. It can be seen that the mitochondrial membrane potential after Au/AgNDs+X-ray treatment is the lowest (Figure 4c). This finding suggests that Au/AgNDs contribute to the improvement of the RT effect by decreasing mitochondrial membrane potential.

DNA double-strand breaks (DSBs) are the most serious incident of ionizing radiation, and failure to repair DSBs leads to chromosomal rearrangements, cell cycle arrest, or cell death.^46,48^ The cell cycle, a series of processes responsible for DNA replication and the production of two daughter cells, plays a crucial role in determining the effectiveness of cell RT. It consists of four phases: G1 phase, S phase, G2 phase, and M phase. Of the four stages, the G2/M phase is particularly sensitive to irradiation.^49,50^ Therefore, biomaterials that can arrest cell cycle of tumor cells in the G2/M phase will enhance the radiosensitivity.^51^ The cell cycle progression was evaluated by flow cytometric determination of the DNA content after propidium iodide (PI) staining. The number of cells in G0/G1, S, and G2/M phases showed that the cells gradually accumulate in G2/M phase following exposure to X-ray radiation (Figure 4d and e). Compared with PBS and AuNDs, Au/AgNDs along with the X-ray radiation cause a higher cell distribution in the G2/M phase and the arrest of the cell cycle mainly in the G2/M phase (Figure 4f). Therefore, Au/AgNDs+X-ray exhibit potent cell killing efficiency and can be used as a promising radiosensitizer.

To further reveal the mechanism of radiosensitivity, DSBs and the repair of DNA inside cancer cells were detected. When DSBs occur, cancer cells are regulated by cell cycle checkpoints, which block cells at a certain time, thus providing sufficient time for the damaged DNA to repair. Dukaew et al. reported eurycomalactone as the radiosensitizer could increase the number of γ-H2AX foci, clog the formation of P53-binding protein 1 (53BP1) foci at the DSB sites, and also dilute the expression of DNA repair protein.^52^

**Figure 5:**
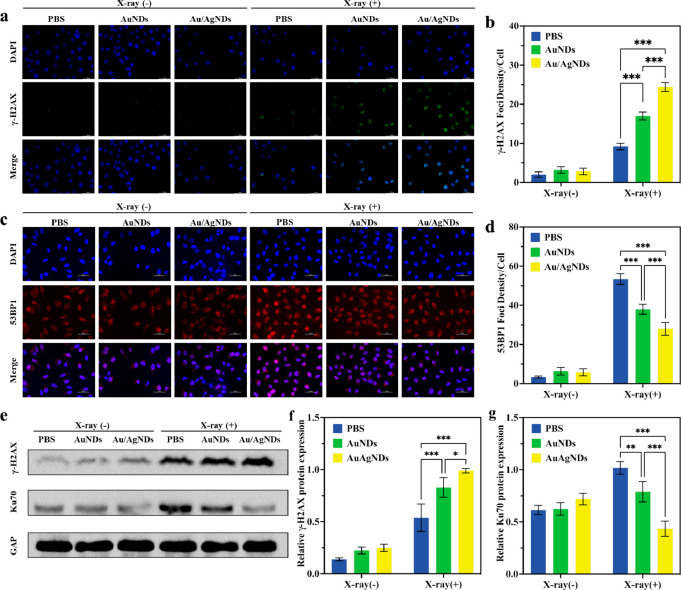
Mechanism of DNA damage and repair. Representative immunofluorescence images of γ-H2AX (a) and 53BP1 (c) in HeLa cells with different treatments, stained with DAPI, γ-H2AX, and 53BP1 for nuclear visualization, DNA double-strand breaks, and the repaired protein of DNA double-strand breaks, respectively (scale bar: 50 μm). The foci number of γ-H2AX (b) and 53BP1 (d) after different treatments. (e–g) Western blotting images and semiquantitative analysis of the expression of γ-H2AX and Ku70 in HeLa cells after different treatments.

The marker γ-H2AX is employed to detect DNA damage following ionizing radiation.^53^ The intensity and number of γ-H2AX foci of PBS, AuNDs, and Au/AgNDs post X-ray irradiation are shown in Figure 5a and b. Faint green fluorescent spots could be seen in the nuclei after single X-ray irradiation. The green fluorescence in the nuclei increased after AuNDs+X-ray treatment. Importantly, more green fluorescence was found in the Au/AgNDs+X-ray group, and the nuclei of some cells were covered with green fluorescence, indicating higher levels of DSBs and severe DNA damage within the nuclei.

53BP1 plays an important role in the repair of DSBs and the maintenance of genomic integrity.^54^ The intensity and number of 53BP1 foci of PBS, AuNDs, and Au/AgNDs post X-ray irradiation are shown in Figure 5c and d. Intense red fluorescent spots could be seen in the nuclei after single X-ray irradiation, indicating ionizing radiation activates the repair process as well as damaging DNA. The red fluorescence decreased with the treatment of AuNDs+X-ray. Moreover, the fewer red fluorescent spots in the nuclei were found in the Au/AgNDs+X-ray group, reflecting the repair of DSBs was inhibited.

Based on the results of immunofluorescence, the proteins of DNA damage and repair pathways were further detected by Western Blotting (Figure 5e). Ku70 is an endogenous nucleoprotein involved in the non-homologous end-joining pathway to repair DSBs.^55^ As shown in Figure 5f and g, AuNDs+X-ray and Au/AgNDs+X-ray increased the protein expression of γ-H2AX and decreased the protein expression of Ku70. Moreover, compared with the AuNDs+X-ray group, γ-H2AX protein expression was further increased and Ku70 protein expression was further decreased in the Au/AgNDs+X-ray group. Overall, the mechanism of Au/AgNDs enhanced RT is to increase the sedimentation of ionizing radiation energy, increase intracellular ROS production, decrease mitochondrial membrane potential, arrest the cell cycle at the G2/M phase, promote DNA damage formation, and inhibit DNA repair, thus significantly improving RT efficacy.

### 2.6 FL/CT Imaging-Guided Precision RT in Vivo

**Figure 6:**
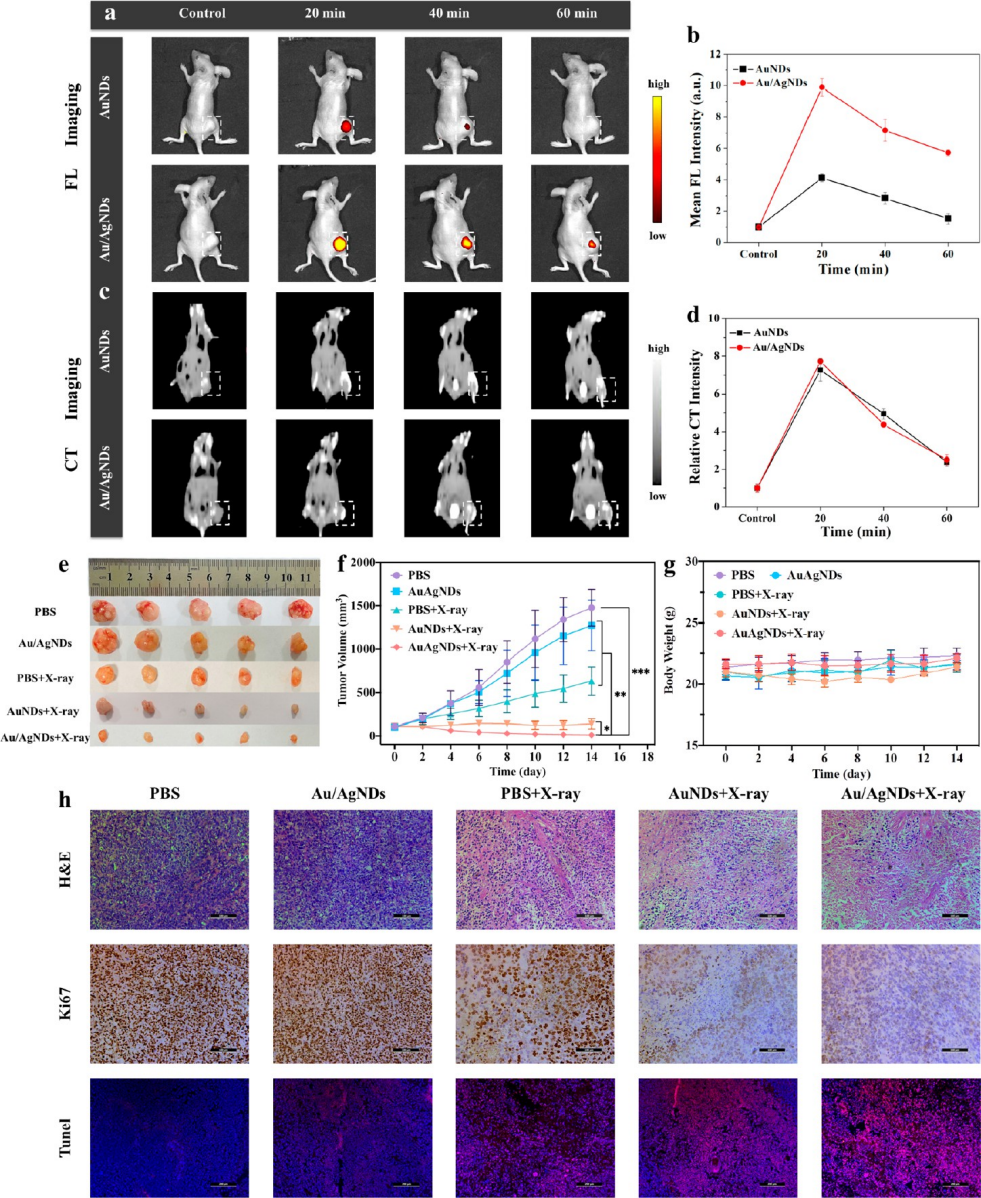
FL/CT dual-mode imaging-guided RT in vivo. (a) FL and (c) CT images of a subcutaneous HeLa tumor-bearing female nude mice model at different time points post intratumoral injection of AuNDs or Au/AgNDs in vivo. The white dashed box in each panel shows the location of the tumor. (b) The FL and (d) CT intensity of AuNDs and Au/AgNDs at different time points post intratumoral injection in vivo. (e) Tumor images, (f) tumor volume, and (g) body weight collected from mice with different treatment groups. The data are exhibited as mean ± SD (*n* = 5). (h) H&E staining (scale bar: 100 μm), Ki67 immunohistochemical staining (scale bar: 100 μm), and TUNEL staining (scale bar: 200 μm) of primary tumor sections from a subcutaneous HeLa tumor-bearing female nude mice model on the 14th day after different treatments.

Due to the difficulty in accurately locating the tumor, ionizing radiation used to kill tumors may damage adjacent normal tissue, causing severe toxicity to the organism. To solve the above problem, the designed Au/AgNDs can serve as a “pilot light” to improve the accuracy of tumor diagnosis by dual-mode imaging. In vivo imaging effects were observed at different time points after the injection of AuNDs or Au/AgNDs. AuNDs showed a weaker FL signal in tumor tissue, which later almost disappeared. Obviously, after injection of the Au/AgNDs, the tumor tissue showed a strong FL signal, which was significantly different from the surrounding normal tissue (Figure 6a and b). The fluorescence signal at the tumor site decreased with time. The fluorescence distribution in resected organs and tumors was observed in the different treatment groups at 2 h post intratumoral injection. Compared with the AuNDs group, tumors in the Au/AgNDs group had a higher sustained fluorescence signal (Figure S11 and Figure S12).

To explore the possibility of Au/AgNDs as contrast agents for clinical tumor diagnosis, CT imaging efficacy was evaluated in vivo. Both AuNDs and Au/AgNDs showed excellent and similar CT imaging performance, and the intensity gradually decreased with time (Figure 6c and d), which was basically consistent with the FL imaging results. Consequently, the combination of FL/CT imaging modalities for tumor detection exhibited high spatial resolution, contrast, and sensitivity, overcoming the limitations of each individual technique. Based on these promising dual-mode imaging effects, the Au/AgNDs holds the potential for precise tumor localization, allowing doctors to accurately locate lesions during treatment, avoid damage to normal tissue, and achieve precise RT.

The RT effect of the nanosystem was further studied using a subcutaneous HeLa tumor-bearing female nude mice model. After the tumor location was determined through dual-mode imaging (FL/CT) in vivo, the mice received different treatments. As shown in Figure 6e,f and Figure S13, all the radiation-treated groups exhibited varying degrees of tumor growth inhibition. Particularly, the Au/AgNDs+X-ray group displayed superior antitumor efficiency compared to the AuNDs+X-ray group, highlighting the excellent radiosensitive properties of Au/AgNDs during RT. To further demonstrate the radiosensitizing effect of the Au/AgNDs, hematoxylin and eosin (H&E) staining, Ki67 immunohistochemical staining, and Terminal Deoxynucleotidyl Transferase-mediated dUTP Nick-End Labeling (TUNEL) staining were performed on the tumor sections after different treatments. H&E results showed that different degrees of tumor cell death were observed after different treatments. The Au/AgNDs+X-ray group showed the largest red-stained structureless necrotic area with loosely arranged tumor cells, indicating that the Au/AgNDs+X-ray group effectively promoted tumor cell death (Figure 6h). Then, different groups of tumor tissue were collected for immunohistochemical staining of the Ki67 antibody. Ki67 is a marker of cell proliferation in solid tumors and certain hematologic malignancies. The level of Ki67-positive staining cells was lower in the Au/AgNDs+X-ray group (Figure 6h). Quantitative analysis of Ki67 positive staining cells showed that the cell proliferation rate of the Au/AgNDs+X-ray group (22.33% ± 2.52%) was lower than that of other groups (Figure S14). Moreover, TUNEL staining of tumor sections was performed after different treatments to further detect the apoptosis of tumor cells (Figure 6h). Vague red fluorescence could be seen after a single X-ray irradiation. The red fluorescence increased after the AuNDs+X-ray treatment. Importantly, more red fluorescence was found in the Au/AgNDs+X-ray group, indicating higher levels of apoptosis. Fluorescence quantitative analysis of TUNEL staining also supported this finding (Figure S15).

In addition, evaluating the in vivo safety of the nanosystem is crucial for its potential clinical use in disease diagnosis and treatment. The weight change is one of the main indicators of organism intoxication. As shown in Figure 6g, there was no obvious body weight change in all groups. Furthermore, histological analysis performed on the 14th day showed no apparent organ damage or abnormalities in the heart, liver, spleen, lung, and kidney of all groups, indicating a lack of systemic or chronic toxicity (Figure S16). In conclusion, our strategy developed an excellent radiosensitizer with the FL/CT dual-mode imaging effect, promising RT effect, and minimal side effects.

### 2.7 FL/CT Imaging-Guided Interstitial Brachytherapy in Vivo

Increasing the local radiation dose of the tumor is the ideal dose requirement of radiation oncology. Several clinical studies^56−58^ have demonstrated that local control and overall survival of patients are strongly correlated with biologically effective doses (BEDs). Current external beam radiotherapy techniques can elevate the tumor dose but are limited by exposure to nearby organs and skin surfaces, posing a risk to healthy tissue. However, in brachytherapy, the radiation source can directly reach the tumor target area. After placement of the implant needle, the dose around the radioactive source drops rapidly. While achieving higher dose deposition in the local tumor area, it also reduces the dose to healthy tissue, organs, and structures surrounding the tumor.

Nevertheless, interstitial brachytherapy is a technically complex and invasive procedure. Accurate needle placement and puncture procedures are challenging without real-time imaging guidance. The current clinical application of CT-guided interstitial brachytherapy will make patients subject to multiple CT scans to change the position of needles frequently, increasing the damage of ionizing radiation to the human body. Therefore, the development of biomaterials that can accurately real-time guide needle placement and improve the efficacy of brachytherapy is urgently needed in clinical practice.

**Figure 7:**
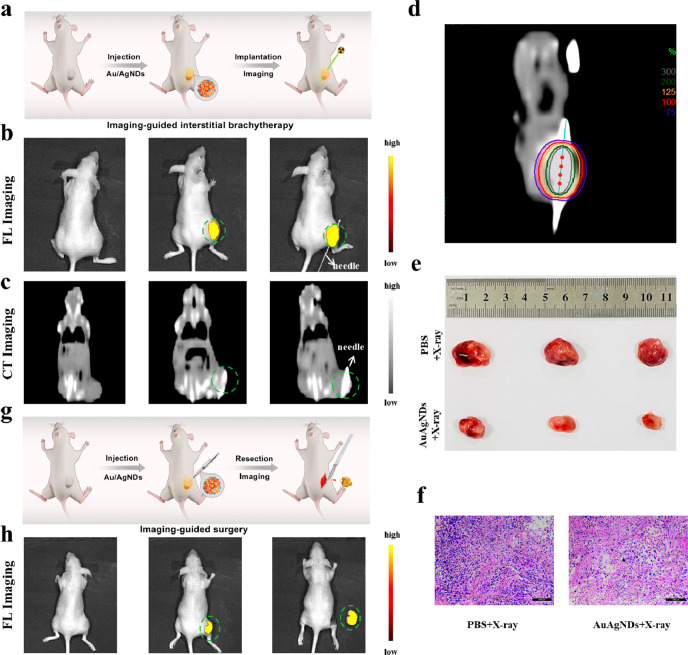
FL/CT dual-mode imaging guided interstitial brachytherapy and FL imaging-guided tumor resection. (a) Schematic illustration of intratumoral injection of Au/AgNDs and imaging-mediated interstitial brachytherapy. (b) Fluorescence and (c) CT imaging of the subcutaneous HeLa tumor-bearing nude mice model post injection of Au/AgNDs and guided interstitial brachytherapy. (d) Dose distribution in a coronal plane for an interstitial brachytherapy plane. (e) Tumor images collected from mice with different treatment groups. (f) H&E staining of primary tumor sections after different treatments (scale bar: 100 μm). (g) Schematic illustration of intratumoral injection of Au/AgNDs and fluorescence-mediated tumor resection. (h) Fluorescence image (control group), FL image of the subcutaneous HeLa tumor-bearing nude mice model post injection of Au/AgNDs, and FL imaging-guided surgical removal of tumor. The green areas represent the tumor.

One promising solution is to combine FL and CT imaging capabilities of Au/AgNDs to guide interstitial brachytherapy, which has the advantages of high spatial resolution, contrast, and sensitivity and can overcome the limitations of each technique. In addition, the excellent FL imaging effect of Au/AgNDs can realize “intraoperative detection” and identify tumor boundaries, providing precise real-time guidance during interstitial brachytherapy. As shown in Figure 7a–c, there is a distinct boundary between the tumor and normal tissues after the injection of Au/AgNDs. The operator can accurately determine the position and depth of the implant needle under real-time guidance of FL and CT imaging. After the location of the insertion needle was confirmed, the dose distribution of the interstitial brachytherapy plan was proposed by the Oncentra physical system. In the coronal plane of dose distribution, it could be seen that the 100% dose profile (red line) basically encompasses the entire tumor region (Figure 7d). Benefiting from the brachytherapy makes the central region of the tumor obtain more than 300% (gray line) of the radiotherapy dose. Meanwhile, with the injection of Au/AgNDs, more radiation doses were deposited in the Au/AgNDs+X-ray group, resulting in a better tumor inhibition effect, and more red-stained structureless necrotic areas could be seen in H&E staining of tumor tissue (Figure 7e and f). In summary, through the use of excellent nanoprobe and radiosensitizer Au/AgNDs, accurate real-time imaging guidance during interstitial brachytherapy has been achieved, improving the therapeutic effect and minimizing the damage to healthy tissue.

### 2.8 FL Imaging-Guided Surgical Resection

In clinical surgery, accurate identification of tumor and normal tissues is significant to prevent recurrence, minimize unnecessary cutting, and avoid damage to normal tissue. FL imaging-guided surgery has received extensive attention in clinical research to achieve accurate tumor resection. Unfortunately, the wider application of this technique has been hindered by the lack of suitable fluorescent probes. To address this limitation, Au/AgNDs with excellent FL properties were designed, and the application in FL-mediated surgical resection was evaluated. As shown in the Figure 7g,h, after the injection of Au/AgNDs, the tumor tissue had obvious fluorescence and a clear boundary with normal tissue. Leveraging the guidance provided by FL imaging, surgeons were able to precisely identify and remove the entire tumor during the surgical procedure. By giving full play to the function of the “pilot light” of Au/AgNDs, it shows promise in enhancing the accuracy and effectiveness of tumor resection in clinical settings.

## 3 Conclusion

In summary, we successfully prepared the multifunctional Au/AgNDs with the ability for real-time dual-mode imaging-guided surgery, enhanced radiotherapy, and brachytherapy of tumors. Compared with AuNDs, Au/AgNDs showed a superior FL imaging effect and improved radiotherapy efficiency, which serves as an excellent nanoprobe and radiosensitizer. The enhanced radiotherapy mechanism of Au/AgNDs is to increase ionizing radiation energy deposition, increase intracellular ROS production, decrease mitochondrial membrane potential, arrest the cell cycle at the G2/M phase, induce tumor cell DNA damage, and inhibit DNA repair, thus significantly improving RT efficacy. The accuracy of detection of cancer was improved through dual-mode imaging (FL/CT), avoiding damage to adjacent normal tissue during X-ray irradiation. In addition, brachytherapy also benefited from Au/AgNDs, as their real-time FL/CT imaging guidance facilitated the precise positioning and depth control of implant needles, and the effect of brachytherapy was also improved. Moreover, due to the excellent FL properties of Au/AgNDs, application in FL-mediated surgical resection was achieved. The “one stone, three birds” strategy indicates that Au/AgNDs have great application potential in the clinical diagnosis and treatment of cancer, paving the way for future advancements in cancer theranostics.

## Supporting Information Available

The detailed synthetic methods, experimental details, supplemental table, FT-IR spectra, high-resolution XPS spectra, EDS spectrum, fluorescence lifetime, fluorescence stability, cell biocompatibility, fluorescence distribution in resected organs and tumors, digital pictures of HeLa tumor-bearing mice after different treatments, quantitative analysis of the Ki67-positive cells and TUNEL staining, and H&E-stained tissue sections of major organs (PDF)
