# Supplementary material for: One Stone, Three Birds: Multifunctional Nanodots as “Pilot Light” for Guiding Surgery, Enhanced Radiotherapy, and Brachytherapy of Tumors
Source: ACS Cent Sci. 2023 Oct 17;9(10):1976–88. doi: 10.1021/acscentsci.3c00994 (PMC10604975; doi:10.1021/acscentsci.3c00994)
Supplement: Supplementary file 1 — oc3c00994_si_001.pdf [file oc3c00994_si_001.pdf]

Supporting Information

One Stone, Three Birds: Multifunctional Nanodots as "Pilot Light" for Guiding Surgery, Enhanced Radiotherapy and Brachytherapy of Tumors

Ze Wang[#], Dongzhou Wang[#], Xiaojun Ren, Zhongshan Liu, Annan Liu, Xingchen Li, Lin Guan, Yannan Shen, Shunzi Jin, Andrei V. Zvyagin, Bai Yang, Tiejun Wang and Quan Lin**

Dr. Z. Wang, Ms. A.N Liu, Ms. X.C. Li, Dr. L. Guan, Ms. Prof. B. Yang, Prof. Q. Lin

State Key Laboratory of Supramolecular Structure and Materials, College of Chemistry, Jilin University, Changchun, 130012, P. R. China

E-mail: linquan@jlu.edu.cn (Q. Lin)

Dr. D.Z. Wang, Dr. X.J. Ren, Prof. Z.S. Liu, Prof. T.J. Wang

Department of Radiation Oncology, the Second Affiliated Hospital of Jilin University, Changchun 130041, P. R. China

NHC Key Laboratory of Radiobiology, School of Public Health, Jilin University, Changchun 130021, P. R. China

E-mail: m13943016598@163.com (T.J. Wang)

Prof. Y.N. Shen, Prof. S.Z. Jin

NHC Key Laboratory of Radiobiology, School of Public Health, Jilin University, Changchun 130021, P. R. China

Prof. A.V. Zvyagin

Australian Research Council Centre of Excellence for Nanoscale Biophotonics, Macquarie University, Sydney, NSW 2109, Australia and Institute of Biology and Biomedicine, Lobachevsky Nizhny Novgorod State University, 603105. Nizhny Novgorod, Russia

1. Experimental Section

1.1. Materials

Silver nitrate ($AgNO_3$) and N, N-dimethylformamide (DMF) were purchased from Beijing Chemical Plant. Tetrachloroauric (III) acid ($HAuCl_4$), 3-mercaptopropionic acid (MPA, 99%) and branched polyethylenimine (PEI) were purchased from Aldrich. N-(3-dimethylaminopropyl)-N'-ethyl carbodiimide hydrochloride (EDC, 98.5%) and N-hydroxysuccinimide (NHS, 98%) were purchased from Aladdin. Hydrazine hydrate ($N_2H_4{\cdot}H_2O$), acetone and trichloromethane were purchased from Sinopharm Chemical Reagent Co., LTD. The water used in the experiment was secondary deionized water. Dulbecco's modified Eagle's medium

(DMEM) was purchased from Gibco. Fetal bovine serum (FBS) was purchased from Gemini. Anti-gamma H2A.X antibody (ab11174) was purchased from Abcam. Anti-GAPDH antibody (BM1623) and Anti-53BP1 antibody (BA2878) were purchased from Boster Biological Technology Co., Ltd. Anti-Ku70 antibody (YK0083) was purchased from ImmunoWay Biotechnology. Anti-Ki67 antibody (bs-2130R) was purchased from Beijing Biosynthesis Biotechnology Co., Ltd. Annexin V-FITC/PI Apoptosis detection kit, cell cycle analysis kit and reactive oxygen species assay kit were purchased from Beyotime Institute of Biotechnology.

1.2. Synthesis of ligand (SH-PEI)

Added 40 mL DMF, 64 mg EDC and 38 mg NHS into a three-necked flask successively, stirred to dissolve. Then 400 mL MPA was added in the flask and stirred for 30 min. 0.6 g PEI was dissolved in 2 mL ethanol and the mixture was slowly added to the flask after fully dissolving. The whole system reacted in nitrogen atmosphere at room temperature for 48 h. After the mixture was evaporated to remove organic matter and concentrated to 3 mL, acetone and trichloromethane were added in a ratio of 3:1. After centrifugation at 8800 rpm for 15 min, the supernatant was removed. The precipitate was dissolved in 5 mL water and stored at -20℃ for later use.

1.3. Synthesis of Au/AgNDs

5 mL water, 200 μL SH-PEI, 250 μL $HAuCl_4$ (50 mM), 25 μL $AgNO_3$ (50 mM), 300 μL $N_2H_4 \cdot H_2O$ were sequentially added into round-bottomed flask. The whole system was heated in oil bath at 80℃ for 4 h. After 12 h of dialysis, the product was stored at room temperature in the dark.

1.4. Characterization

The fluorescence spectra were measured using Shimadzu RF-5301 PC fluorescence spectrometer. The UV-visible absorption spectra were obtained by Lambad 800 UV-visible spectrophotometer. X-ray photoelectron spectra were measured by VG ESCALAB MKII spectrometer. The Zeta potential of the samples was measured by Zetasizer Nano ZS particle size analyzer. Fourier transform infrared (FTIR) spectra of the samples were measured by Nicolet Avatar 360 Fourier

transform infrared spectrometer. Transmission electron microscope (TEM) photos were taken by JEOL TECNAI F20 field emission electron microscope with the operating voltage of 200 kV. Confocal microscope photos were captured with Olympus Fluoview FV1000 confocal microscope.

1.5. Cell lines and cell culture

HeLa cells were obtained from Boster Biological Technology co.ltd. L929 cells were obtained from NHC Key Laboratory of Radiobiology. They were cultured in DMEM with 10% fetal bovine serum and 1% penicillin-streptomycin, and cultured in the incubator with suitable environment at 37°C and 5% CO_2.

1.6. Cell uptake

HeLa cells were plated at 1×10^5 cells per well in confocal dishes overnight. After the cells were cultured with AuNDs or Au/AgNDs for another 12 h, it was detected by CLSM.

1.7. Cellular viability assay

The percentage of the viable cell was detected by CCK-8 assay. HeLa cells were plated at 2,000 cells per well and L929 cells were plated at 3,000 cells per well in 96-well plates overnight. After the HeLa cells were cultured with AuNDs or Au/AgNDs for another 12 h or 24 h, and the L929 cells were cultured with AuNDs or Au/AgNDs for another 24 h, 10 μL of the CCK-8 stock solution was added to each well and the plate was incubated for 2 h at 37 °C. Then it was measured by BioTek Epoch ultramicroscopic microplate spectrophotometer at a wavelength of 450 nm.

1.8. Colony formation assay

HeLa cells were plated at 500 cells per well in 6-well plates and allowed to attach for 24 h. Then the cells were cultured with AuNDs or Au/AgNDs at the indicated concentration for 12 h. At 12 h they were irradiated at 0, 2, 4, 6, 8 Gy, and then further cultured for 10-14 d separately.

1.9. Intracellular ROS and mitochondrial membrane potential assay

HeLa cells were plated at 2×10^5 cells per well in 6-well plates overnight. Then the cells were cultured with AuNDs or Au/AgNDs at the indicated concentration for 12 h. At 12 h they were irradiated with/without 6 Gy of X-ray. 2 h after irradiation,

the cells were washed and incubated with 10 μM DCFH-DA for further 20 min or JC-1 for 30 min, and then the fluorescence images were acquired by fluorescence microscope (Cytation 3).

1.10. Flow cytometry assay

HeLa cells were plated at 6×10^5 cells per well in 6-well plates overnight. Then the cells were cultured with AuNDs or Au/AgNDs at the indicated concentration for 12 h. At 12 h they were irradiated with/without 6 Gy of X-ray. The cells were further cultured for 24 h.

1.11. Cell apoptosis assay

The cells were prepared for flow cytometry. The samples were stained by Annexin V-FITC/PI apoptosis detection kit following the manufacturer's protocol.

1.12. Cell cycle assay

The cells were prepared for flow cytometry. They were fixed with ice-cold 70% ethanol in distilled water and stained with the PI following the manufacturer's protocol of cell cycle analysis kit.

1.13. Western blotting analyses

HeLa cells were seeded at 1×10^6 cells in 10 cm dishes and allowed to attach for 24 h. The cells were cultured with AuNDs or Au/AgNDs at the indicated concentration for 12 h. At 12 h they were irradiated with/without 6 Gy of X-ray. The proteins were extracted from cells with RIPA and phenylmethylsulfonyl fluoride (PMSF). After quantification by BCA protein assay kit, samples were mixed with 5X SDS loading buffer and separated by sodium dodecyl sulfate polyacrylamide gel electrophoresis (SDS-PAGE). After transfer, the printing membranes were blocked with skimmed milk for 90 min and incubated with primary antibody at 4°C overnight. Afterwards, the membrane was washed 3X 10 min in TBST, and then the membrane was incubated with secondary antibody for 2 h. The membrane was washed again 3X 10 min in TBST and enhanced chemiluminescence was applied to visualization.

1.14. Immunofluorescence assay

HeLa cells were plated at 1×10^5 cells per well in confocal dishes overnight. After treatment with/without nanoparticles and X-ray, HeLa cells were washed 3X 3 min

with cold PBS and fixed with 4% para formaldehyde at room temperature for 15 min, permeabilized with 0.1% Triton X-100 at room temperature for 5 min, blocked with 5% BSA for 1 h, and stained with primary antibody (Anti-gamma H2A.X and Anti-53BP1) at 4°C overnight. Afterwards, the cells were washed 3X 3 min in TBST, and then the cells were incubated with fluorescent secondary antibody for 1 h. After staining with DAPI for another 20 min, the cells were observed under fluorescence microscopy (Lecia DM4000B).

1.15. In vivo imaging

The HeLa tumor-bearing female nude mice received intraperitoneal injection of pentobarbital sodium (40 mg/kg) for anaesthetization. The mice were intratumorally injected with AuNDs or Au/AgNDs (10 mg/kg). Fluorescence imaging and CT imaging were performed at 0, 20, 40, 60 min. After AuNDs or Au/AgNDs injected for 2 h, the mice were euthanized, and main organs and tumors were isolated for fluorescence imaging (PerkinElmer IVIS Lumina LT III). Finally, the fluorescence intensity was evaluated.

1.16. In vivo antitumor therapy

Female nude mice (4-5 weeks old) were purchased from Beijing HFK Bio-Technology co.,LTD (Beijing, China). All animal experiments were conducted according to the Laboratory Animal-Guideline for ethical review of animal welfare and Institutional Animal Care and Use Committee of Jilin University (No. SY202302027). The mice received intraperitoneal injection of pentobarbital sodium (40 mg/kg) for anaesthetization before treatments. HeLa cells were suspended in PBS at 1×10^7/mL, and 100 μL cell suspension was subcutaneously injected into the right flanks of mice. The sizes (lengths and widths) of the tumors were measured by a digital caliper every two days, and the volumes of the tumors were calculated by the following equation: Tumor volume (mm^3) = 1/2 × length ×$width^2$. When the tumor size reached 100 mm^3, all mice were randomly divided into five groups (n=5). Then, these mice were intratumorally injected with PBS, AuNDs or Au/AgNDs (10 mg/kg) under general anesthesia. After 20 min, X-rays at 6 Gy were exposed to the corresponding group of mice. The tumors sizes were monitored every 2 days. At 14

days posttreatment, all the animals were sacrificed by euthanasia. The main organs (heart, liver, spleen, lungs and kidney) were harvested and fixed for H&E staining. The tumors were harvested and fixed for pathological examination (H&E, Ki67 and TUNEL staining).

1.17. In vivo imaging-guided interstitial brachytherapy

Under general anesthesia, the HeLa tumor-bearing female nude mice were intratumorally injected with Au/AgNDs (10 mg/kg). Fluorescence imaging and CT imaging were performed at 20 min. Guided by real-time fluorescence and CT imaging, the implant needle was positioned at the appropriate location within the tumor. Then the CT images were transferred to the Oncentra physical system. Subsequently, the target area was contoured, the treatment plan was made and the dose was assessed. After passing the above process, the brachytherapy was approved and implemented. At 7 days posttreatment, all the animals were sacrificed by euthanasia. The tumors were harvested and fixed for H&E staining.

1.18. In vivo imaging-guided surgical resection

Under general anesthesia, the HeLa tumor-bearing female nude mice were intratumorally injected with Au/AgNDs (10 mg/kg). Fluorescence imaging was performed at 20 min. With FL imaging, a distinct tumor boundary could be seen and the tumor was removed.

1.19. Statistical analysis

The experimental data was indicated as mean ± SD. Student's *t*-test was used for statistical analysis between two groups, and one-way ANOVA was used for statistical analysis between multiple groups. The statistical significance was speculated at a value of $^*p < 0.05$, $^{**}p < 0.01$, $^{***}p < 0.001$.

2. Supplementary Figures

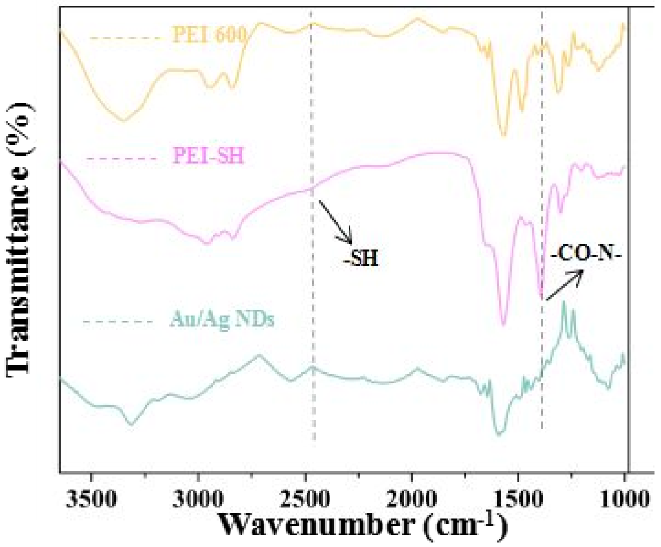

Figure S1. FT-IR spectra of PEI, SH-PEI and Au/Ag NDs.

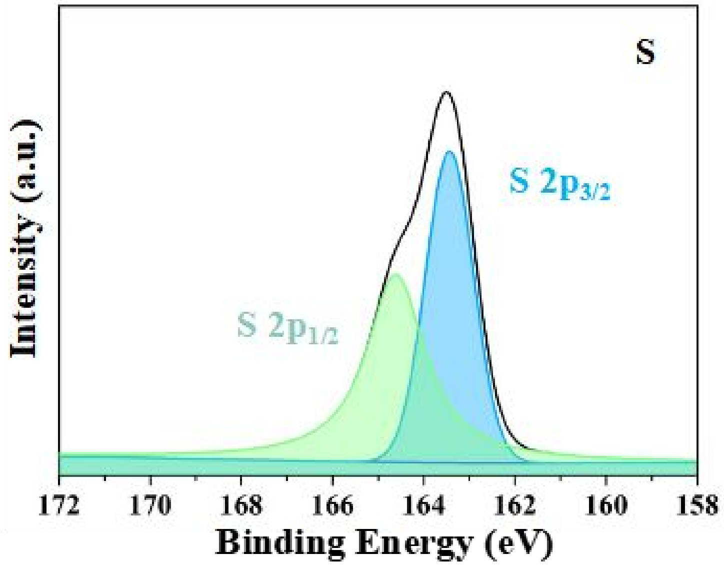

Figure S2. High-resolution XPS spectra of S 2p of Au/AgNDs.

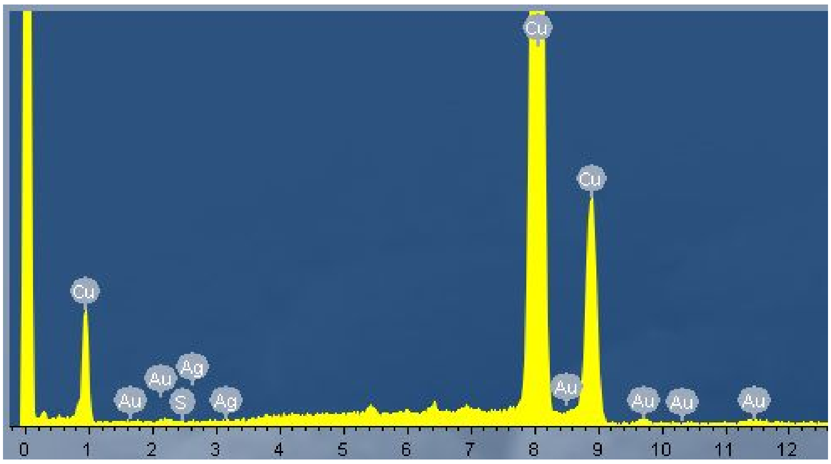

Figure S3. Energy-dispersive spectroscopy (EDS) spectrum indicating the coexistence of Au and Ag.

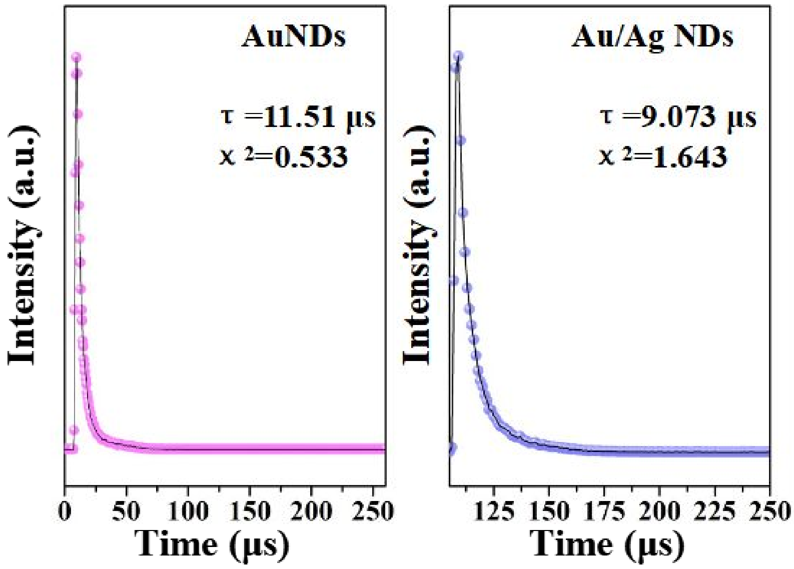

Figure S4. Fluorescence lifetime curves of AuNDs and Au/Ag NDs.

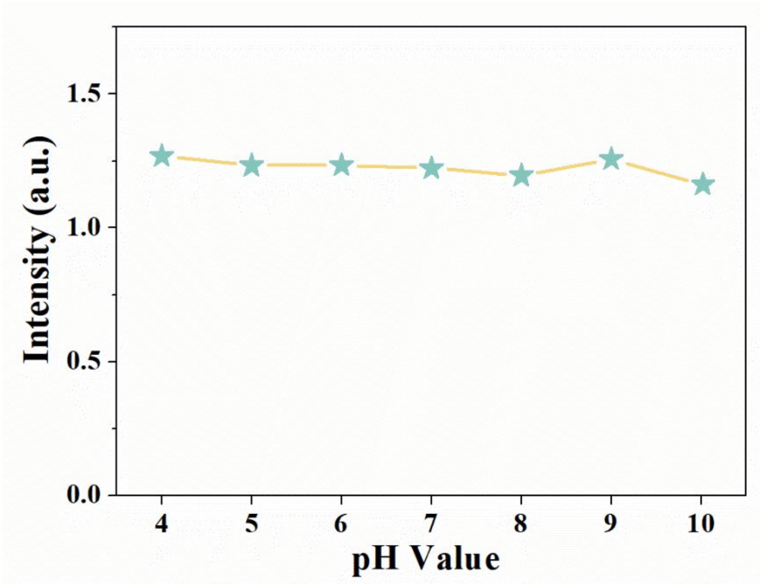

Figure S5. Fluorescence intensity of Au/AgNDs at different pH value.

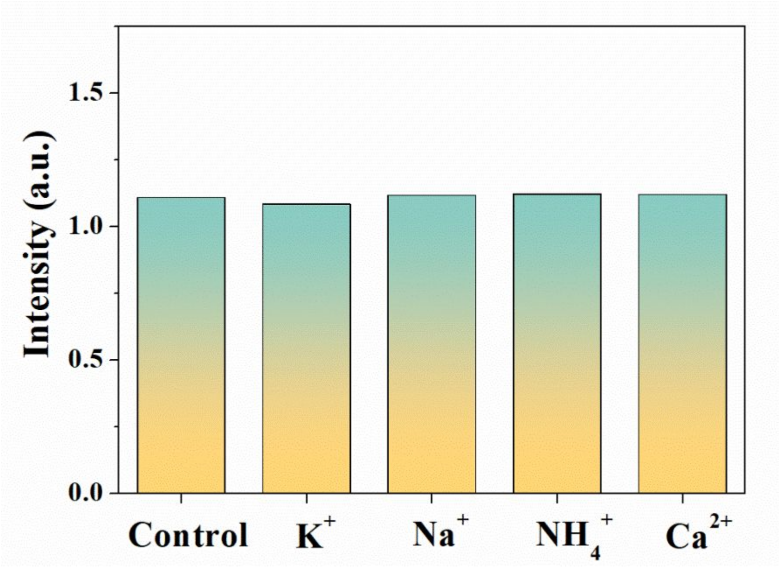

Figure S6. Fluorescence intensity of Au/AgNDs at high concentration of interfering ions (K^+, Na^+, NH_4^+, Ca^{2+}, 200 mM of each ion).

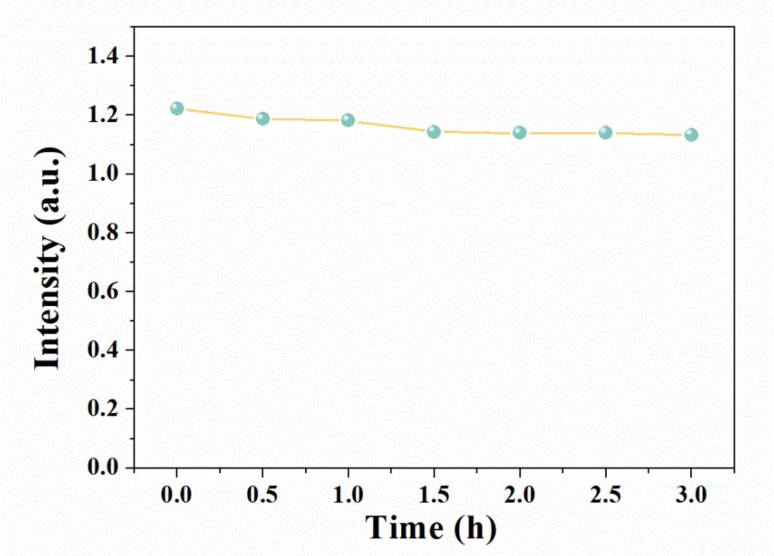

Figure S7. Fluorescence intensities of Au/AgNDs exposed under UV light for various time spans.

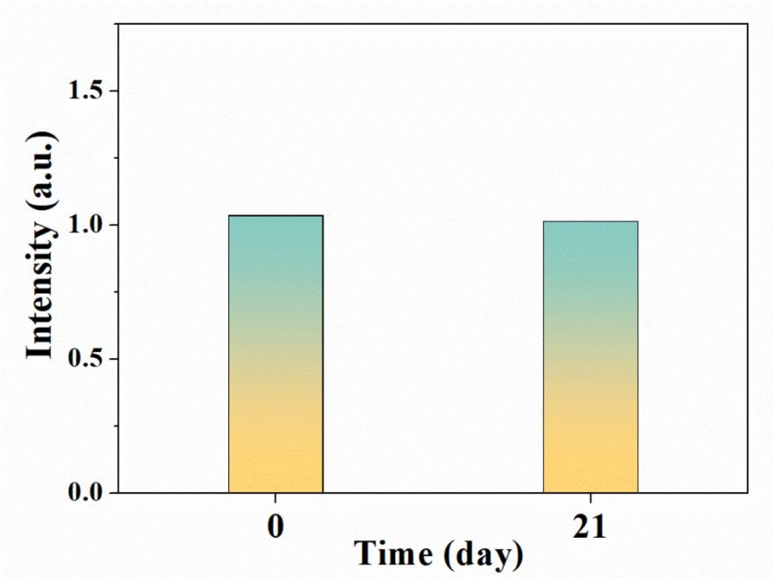

Figure S8. Fluorescence intensity of Au/AgNDs storing under normal condition for 21 days.

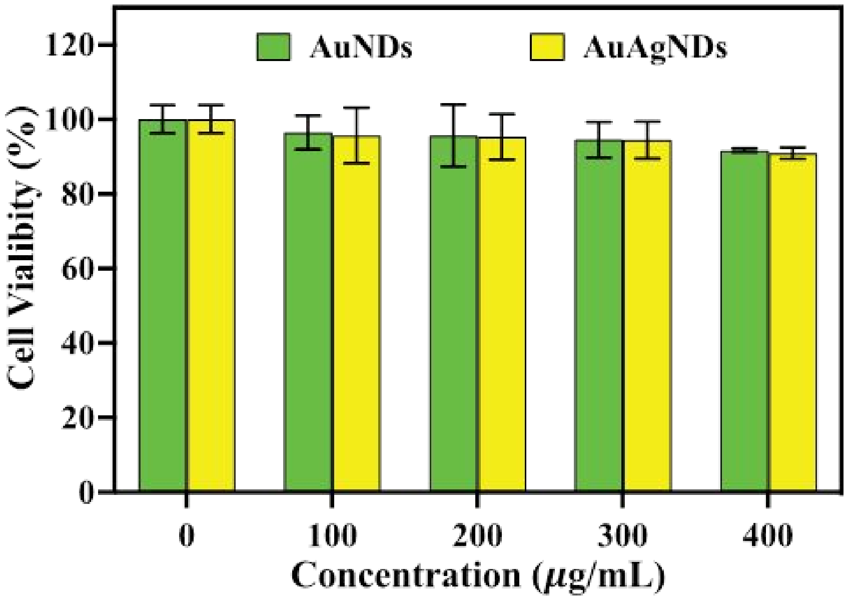

Figure S9. Effect of AuNDs and Au/AgNDs on the viability of L929 cells at different concentrations for 24 h.

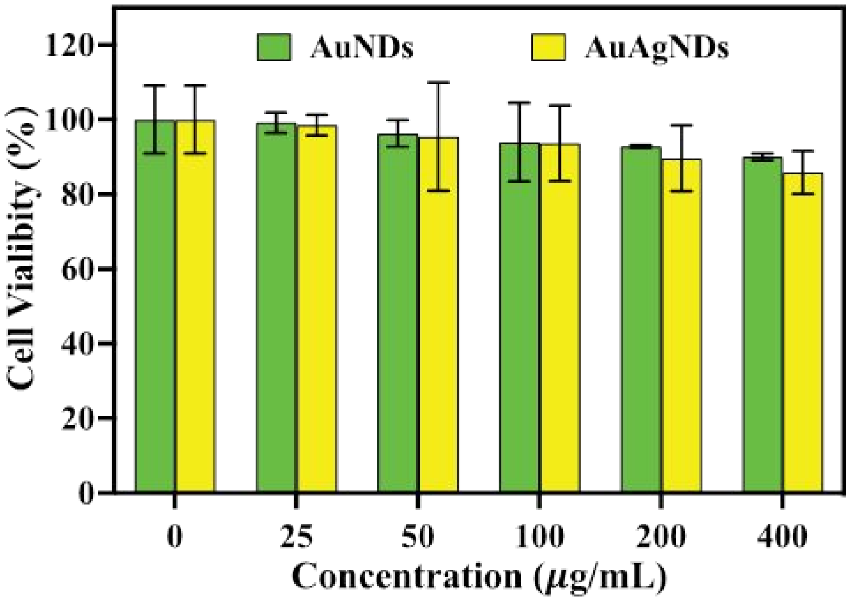

Figure S10. Effect of AuNDs and Au/AgNDs on the viability of HeLa cells at different concentrations for 12 h.

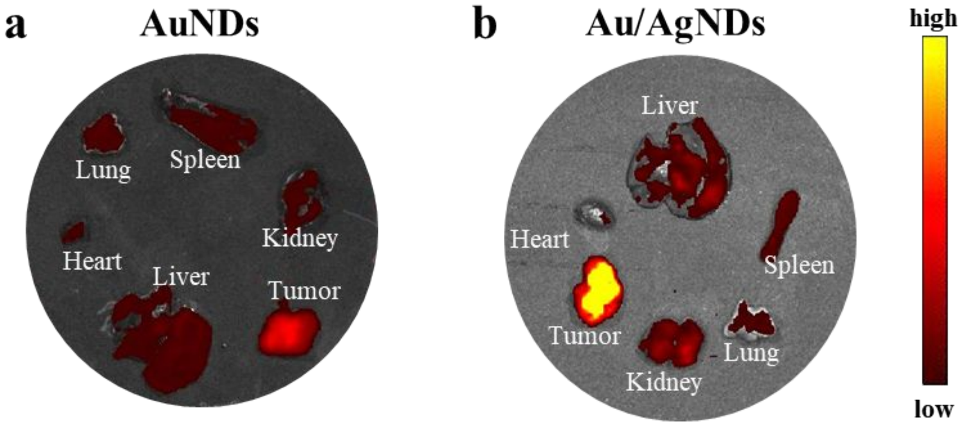

Figure S11. (a)-(b) Fluorescence distribution in resected organs and tumors in different treatment groups.

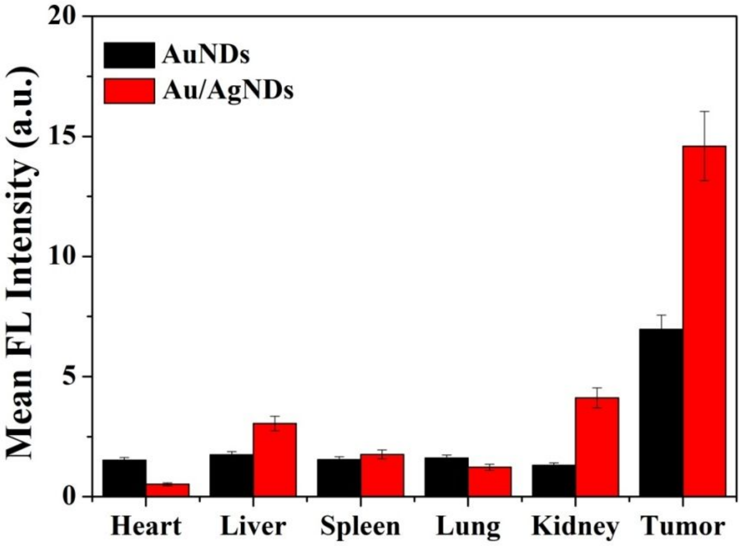

Figure S12. The quantitative analysis of the fluorescence intensity in excised organs and tumors for different treatment groups.

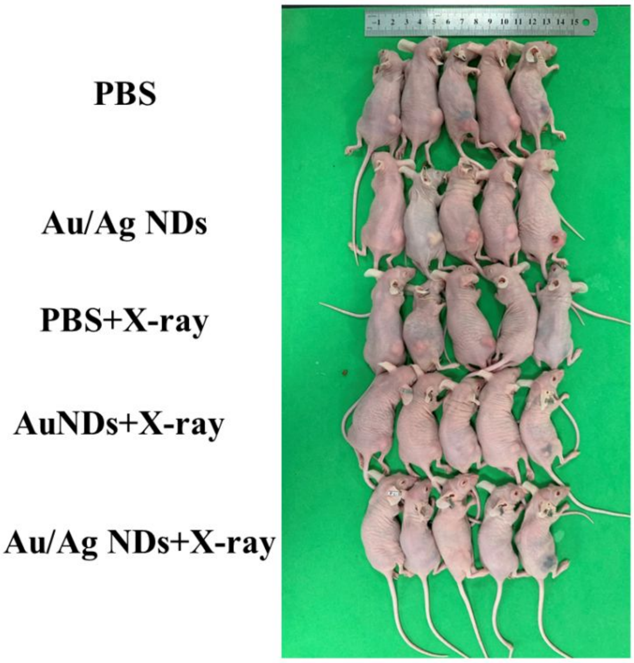

Figure S13. Digital pictures of HeLa tumor-bearing mice after different treatments over 14 days.

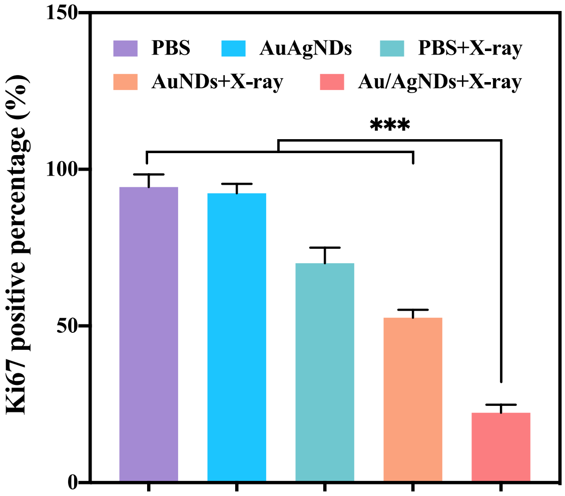

Figure S14. Quantitative analysis of the Ki67-positive cells in tumor sections after different treatments.

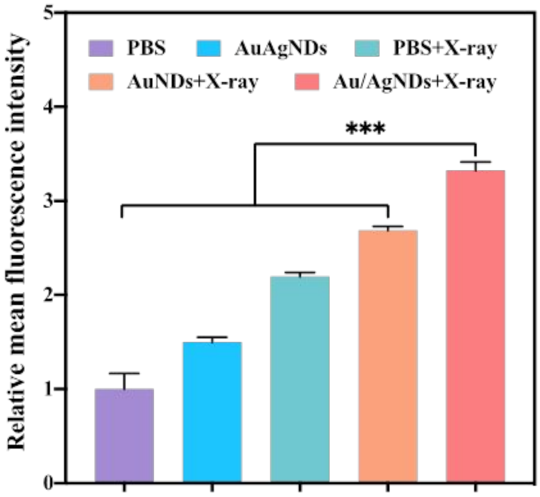

Figure S15. Quantitative analysis of the TUNEL staining of primary tumor sections after different treatments.

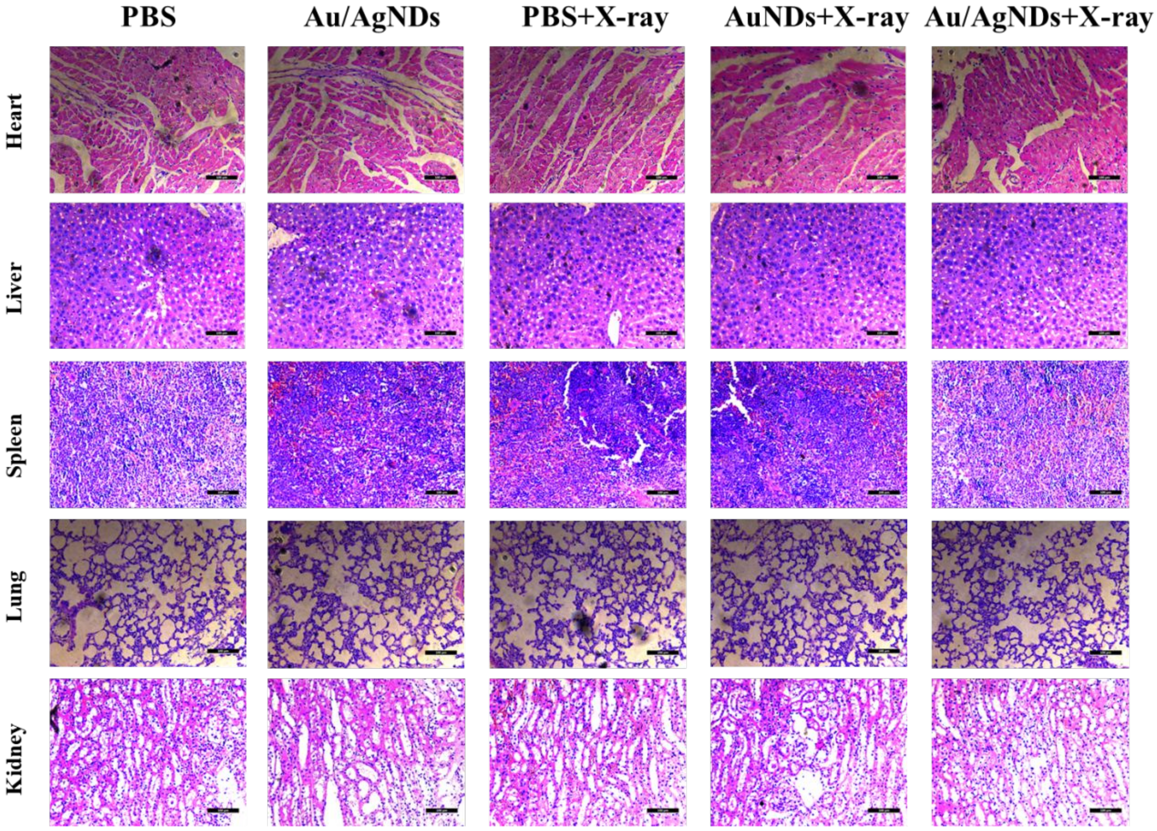

Figure S16. H&E-stained tissue sections of major organs (heart, liver, spleen, lung and kidney) of mice in different treatment groups (Scale bar: 100 μm).

Table S1. The D_0, n, D_q, SF_2 and R^2 value of single-click multi-target model.

Group	D_0	n	D_q	SF_2	R^2
PBS+X-ray	2.011	3.077	2.260	0.758	0.9972
AuNDs+X-ray	1.616	3.076	1.816	0.652	0.9981
Au/AgNDs+X-ray	1.194	2.911	1.276	0.453	0.9998
